# Risk stratification integrating genetic data for factor VIII inhibitor development in patients with severe hemophilia A

**DOI:** 10.1371/journal.pone.0218258

**Published:** 2019-06-13

**Authors:** Delphine Bachelet, Thilo Albert, Cyprien Mbogning, Signe Hässler, Yuan Zhang, Stephan Schultze-Strasser, Yohann Repessé, Julie Rayes, Anna Pavlova, Behnaz Pezeshkpoor, Kerstin Liphardt, Julie E. Davidson, Agnès Hincelin-Méry, Pierre Dönnes, Sébastien Lacroix-Desmazes, Christoph Königs, Johannes Oldenburg, Philippe Broët, on behalf of the ABIRISK consortium

**Affiliations:** 1 CESP, INSERM UMR 1018, Faculty of Medicine, Paris-Sud University, UVSQ, Paris-Saclay University, Villejuif, France; 2 Institute of Experimental Haematology and Transfusion Medicine, University Clinic Bonn, Bonn, Germany; 3 University Hospital Frankfurt, Goethe University, Department of Pediatrics, Molecular Haemostasis and Immunodeficiency, Frankfurt am Main, Germany; 4 CHU Caen, Hématologie Biologique, Caen, Caen, France; 5 Sorbonne Universités, UPMC Univ Paris 06, INSERM, Université Paris Descartes, Sorbonne Paris Cité, UMR_S 1138, Centre de Recherche des Cordeliers, Paris, France; 6 GlaxoSmithKline, Uxbridge, Middlesex, United Kingdom; 7 Sanofi, Chilly-Mazarin, France; 8 SciCross AB, Skövde, Sweden; 9 AP-HP, Paris-Sud University Hospitals, Villejuif, France; Institut d'Investigacions Biomediques de Barcelona, SPAIN

## Abstract

Replacement therapy in severe hemophilia A leads to factor VIII (FVIII) inhibitors in 30% of patients. Factor VIII gene (F8) mutation type, a family history of inhibitors, ethnicity and intensity of treatment are established risk factors, and were included in two published prediction tools based on regression models. Recently investigated immune regulatory genes could also play a part in immunogenicity. Our objective is to identify bio-clinical and genetic markers for FVIII inhibitor development, taking into account potential genetic high order interactions. The study population consisted of 593 and 79 patients with hemophilia A from centers in Bonn and Frankfurt respectively. Data was collected in the European ABIRISK tranSMART database. A subset of 125 severely affected patients from Bonn with reliable information on first treatment was selected as eligible for risk stratification using a hybrid tree-based regression model (GPLTR). In the eligible subset, 58 (46%) patients developed FVIII inhibitors. Among them, 49 (84%) were “high risk” F8 mutation type. 19 (33%) had a family history of inhibitors. The GPLTR model, taking into account F8 mutation risk, family history of inhibitors and product type, distinguishes two groups of patients: a high-risk group for immunogenicity, including patients with positive HLA-DRB1*15 and genotype G/A and A/A for IL-10 rs1800896, and a low-risk group of patients with negative HLA-DRB1*15 / HLA-DQB1*02 and T/T or G/T for CD86 rs2681401. We show associations between genetic factors and the occurrence of FVIII inhibitor development in severe hemophilia A patients taking into account for high-order interactions using a generalized partially linear tree-based approach.

## Introduction

For severe hemophilia A (HA) patients, the current standard of care includes regular prophylactic infusions of factor VIII (FVIII) products in order to prevent spontaneous bleeds or on demand infusions to treat bleeds. The main concern nowadays is the development of inhibitors that neutralize the activity of the FVIII molecule, which occurs mainly in the first 20 days of exposure for approximately 30% of the patients. In this context, the search for risk factors for immunogenicity of FVIII products is of primary concern in order to understand the mechanisms leading to the development of inhibitors and ultimately to prevent their development.

Many factors (patient-, disease- or product-related) could influence the potential risk for immunogenicity of biotherapeutics, but the relative contributions of these factors to the development of neutralizing antibodies is currently not completely understood. Several risk factors of inhibition against FVIII products are well recognized, such as factor VIII gene (F8) mutation type, a family history of inhibitors, ethnicity, intensity [1], but others are still under debate. Concerning the product type, it was shown in a randomized prospective trial (SIPPET) that patients treated with plasma-derived factor VIII containing von Willebrand factor had a lower incidence of inhibitors than those treated with recombinant factor VIII [2].

In this search for risk factors of immunogenicity, the genetic diversity of immune regulatory genes, which may have a role in the immunogenicity of FVIII products, has been the subject of recent investigations [3,4]. Table 1 gives a summary of recently published results, which have focused on specific HLA alleles and immune genes.

**Table 1:** Summary of studies finding statistically significant associations between genetic factors evaluated in the present study and inhibitor development in severe hemophilia A.

Genetic factor	Author, year	Country	# Patients—total and with inhibitors (inh+)	Haplotype / Allele / SNP (rs)	Results	Comments
HLA	Oldenburg, 1997 [5]	Germany	71 patients, 29 inh+	DQA1*0102	OR = 2.2 n.s.	Haplotype DQA1*0102, DQB*0602, DR15 occurred more often in inhib+
				DR15	OR = 2.2 n.s.	
	Hay, 1997 [6]	United Kingdom	176 patients, 52 inh+	DQA1*0102	OR = 3.1 [1.0–10.1]	Analyses also stratified on mutation type (intron 22 inversion vs others). DRB*1501, DQB1*0602, DQA1*0102 is an established haplotype
	Pavlova, 2009 [3]	Germany	260 patients, 130 inh+	DRB1*15	OR = 1.99 [1.21–3.25]	Inh+ and inh- patients were matched by mutation type Haplotypes also studied
				DQB1*0602	OR = 1.99 [1.15–3.40]	
	De Barros, 2012 [7]	Brazil	122 patients, 36 inh+	DRB1*14	OR = 4.87 [1.14–24.41] Re-calculated	Not only severe HA patients
	Pergantou, 2013 [8]	Greece	52 patients, 28 inh+	DRB1*01	OR = 10.9 [1.3–93.9]	
				DQB1*05:01	OR = 12.8 [1.5–109.3]	
				DRB1*11	OR = 0.2 [0.06–0.6]	
				DQB1*03	OR = 0.15 [0.04–0.55]	
IL-10	Astermark, 2006 [9]	MIBS group: several European countries and Toronto, Canada	siblings. 60 unrelated families, 124 patients, 63 inh+	allele 134 in the IL-10G microsatellite	OR = 5.4 [2.1–13.7]	Not only severe HA patients
	Pavlova, 2009 [3]	Germany	260 patients, 130 inh+	-1082 G>A (rs1800896) G vs A	OR = 1.59 [1.12–2.24]	Haplotypes with TNFA also studied
	Lozier, 2011 [10]	48 centers in North America and Europe	915 Caucasian patients, 282 inh+	six SNPs (contains -1082 G>A)	p<0.05	Interaction with HIV status: the global effect of IL-10 haplotypes on inhibitors was stronger in HIV-positive subjects TNFA, CTLA4 also studied but no significant association with inhibitor status
				CTT haplotype at rs6667202, rs4072226, rs4072227	OR = 1.23 [1.01–1.50]	
	Pinto, 2012 [11]	India	120 patients, 50 inh+	-1082 G>A (rs1800896)	OR = 1.85 [0.94–3.70]	Haplotype analysis with two other IL-10 SNPs (rs1800871 and rs1800872)
				allele G vs A	Re-calculated	
	Pergantou, 2013 [8]	Greece	52 patients, 28 inh+	Haplotype -1082G>A, -819C>T, -592C>A ACC or ATA homozygotes vs others	OR = 4.7	
TNFA	Astermark, 2006 [12]	MIBS group: several European countries and Toronto, Canada	siblings. 60 unrelated families, 124 patients, 63 inh+	-308 G>A (rs1800629) A/A vs others	OR = 19.2 [2.4–156.5]	
	Pavlova, 2009 [3]	Germany	260 patients, 130 inh+	-308 G>A (rs1800629) A/A vs others	OR = 4.76 [1.00–22.47]	3 other TNFA SNPs also analyzed but no significant association with inhibitor status
	Pinto, 2012 [11]	India	120 patients, 50 inh+	-308 G>A (rs1800629)	NS—n.a.	4 other TNFA SNPs also analyzed but no significant association with inhibitor status
				rs1799724 C/T vs others	OR = 3.19 [1.27–7.99]	
CTLA4	Astermark, 2007 [13]	MIBS group: several European countries and Toronto, Canada	siblings. 60 unrelated families, 124 patients, 63 inh+	-318 C>T (rs5742909) allele T vs C	OR = 0.3 [0.1–0.8]	
	Pavlova, 2009 [3]	Germany	260 patients, 130 inh+	CT60 A>G (rs3087243) allele A vs G	OR = 0.72 [0.51–1.02]	2 other CTLA4 SNPs also analyzed
HMOX1	Repesse, 2013 [14]	France and Germany	362 patients, 99 inh+	(GT) repeats:		
				<21 = S; 21–29 = M;>30 = L		
				LL/LM/LS vs others	OR = 2.21 [1.30–3.76]	
MAPK9	Astermark, 2013 [4]	HIGS combined cohorts from Europe, North America, Latin America, South Africa	833 patients	rs4147385	OR = 2.03 [1.48–2.78]	Results of genetic metaanalysis (Illumina iSelect 14626 SNPs: inflammatory and immune genes)
CD32—FCGR2A	Eckhardt, 2014 [15]	MIBS group: several European countries and Toronto, Canada	85 Caucasian patients 44 unrelated families	rs1801274		5 other FCGR SNPs also analyzed but no significant association with inhibitor status
				CT vs CC	OR = 1.8 [1.1–2.9]	
				TT vs CC	OR = 3.3 [1.2–8.7]	

For the purpose of risk prediction, researchers have also investigated the potential of classic regression models for quantifying an individual’s risk using a clinical scoring system. In this context, two studies have investigated immunogenicity risk factors and proposed prediction scores [16,17]. The first study was based on 332 previously untreated patients (PUPs) born in the 90s and the method was validated on an external cohort of 64 patients. Predictive factors were positive family history, high risk of F8 mutation and initial intensive treatment [16]. The second study included 825 PUPs born 1990–2007 and was validated internally. The predictors were the same as in the first study with an improved definition of treatment intensity combining dose and duration of intensive treatment [17].

Our main objective was to identify bio-clinical and genetic markers associated with the development of FVIII inhibitor taking into account potential genetic high order interactions. The reason for also considering non-linear approaches is that high order interactions between genetic variants are to be expected. We thus decided to consider a recursive, partitioning model (tree-based model) which is better suited for exploring such interactions than standard regression models.

The increasing interest in using regression tree-based models for studying hemophilia A has been clearly discussed by Henrard et al. in a recent article [18]. Compared to a generalized linear model (linear, logistic), the authors underlined the fact that regression trees can easily cope with interactions and identify them in the final model. Such interactions are of primary concern when dealing with HLA markers, and the use of regression trees could provide meaningful subdivisions of HLA markers that are in strong linkage disequilibrium [19].

In this research, we used a hybrid strategy that combined a linear structure for the variables that were not expected to interact with each other and a tree-based structure for those that were expected to interact. In practice, the tree-based model is considered in a second step after adjustment on the variables that are found to be significant in the multivariate logistic model. The main aim is to be able to detect important genetic interactions that could have been overlooked by the logistic model. This tree-based model is a hybrid multivariate structure called Generalized Partially Linear Tree-based Regression (GPLTR) that integrates the advantages of generalized linear regression and tree-based models. The linear part is used to model the effect of the known classic risk factors and the nonparametric tree part is used to capture the distributional shape of the data using the complex joint effects of multiple genetic explanatory variables.

The analyses were performed on cohorts of hemophilia A patients with a long follow-up from two German centers that are reference laboratories for anti-FVIII antibody testing. From this dataset, we evaluated the combined role of clinical and genetic factors in the immunogenicity of FVIII products on a selected population of severe HA PUPs in order to reduce the magnitude of any potential confounding variables associated with therapeutic changes over time.

## Materials and methods

### Design and study population

The population eligible for inclusion in this historical cohort analysis was selected from two German sites (Bonn and Frankfurt) under the leadership of the ABIRISK EU-consortium. For Bonn, follow-up data was collected prospectively for HA patients (children and adults) treated with FVIII products since 1967. The Bonn database was created in 1978 and became an electronic system in 1990. For Frankfurt, data for HA patients having entered services since 1979 was collected in an electronic database created in 2005. For both databases, patients could have been treated previously elsewhere before registering at the current sites.

In all, 593 patients with severe HA from the Bonn database and 79 from the Frankfurt database were included in the present study. Data were fully anonymised prior to access for the analysis.

### Genotyping information

HLA class II (HLA-DRB1, HLA-DQB1) typing was performed by PCR sequence-specific primer (SSP) methodology (Olerup SSP AB; Invitrogen Ltd, One Lambda Inc) following the manufacturer’s protocol.

SNP variants for IL-10 1082A>G (rs1800896), CTLA4 CT60A>G (rs3087243), TNF 308G>A (rs1800629), CD32 500 A>G (rs1801274), MAPK9 (rs4147385) were genotyped. For the CD86 gene, four biallelic SNPs were investigated: rs2715267 in the promoter region, rs2681417 in the exon 4 region, rs1129055 in the exon 7 region and rs2681401 in the untranslated transcribed region (UTR). These four biallelic SNPs were selected as candidate SNPs for the analysis, since a team from the ABIRISK consortium has previously shown that antigen presenting cells are activated when exposed to specific FVIII concentrates and that this activation correlates with CD86 expression levels [20].

All these biallelic polymorphisms were detected by PCR amplification and direct sequencing.

Regarding HMOX-1, the 5’-flanking region of the HO-1 gene containing the (GT)_n_ dinucleotide repeat was amplified as described elsewhere [21]. Each repeat number was calculated with GeneMapper (Applied Biosystems). To confirm the size of (GT)_n_ repeats, selected samples were subjected to a sequence analysis.

### Candidate variables

Data on patient and disease characteristics, such as the mutation type of the F8 gene classified as “high risk” (large deletions, nonsense mutation, intron 22 or intron 1 inversions) versus “low risk” (small deletions/insertions of <200 base pairs, missense mutations, and other mutations including splice site defects), family history of factor VIII inhibitors, and blood group, were considered in the present study.

The type of the first FVIII product (plasma-derived versus recombinant product) was available for a restricted sub-cohort of patients who were first treated in the center hosting the database after its creation, or who had a reliable documentation of their first treatment if they were treated elsewhere. For readability, this sub-cohort is referred to by the term “well-documented” population.

The genetic factors mentioned in the previous section were also considered as potential markers.

Data was standardized into a common format and entered into the ABIRISK tranSMART database, which integrates immunogenicity data from a number of European countries in a well-structured format. The data is harmonized across different cohorts and the format is based on the CDISC standards (http://www.cdisc.org/). Where no previous variable description can be found in CDISC, a local variable description is used. Data was prepared by data custodians using a data load plan describing the variable semantics and format. Anonymized data was uploaded into the ABIRISK database, which is based on the tranSMART platform [22]. The tranSMART platform is an open-source knowledge management platform for translational science, supported by a large number of organizations (http://www.transmartfoundation.org/).

### Outcome definition

The inhibitor status of hemophilia patients was defined as being positive (inhibitor patient group, inh+) or negative (control patient group, inh-) from laboratory results of current Bethesda assays, or historical results for earlier patients. Positivity in laboratories is defined by two consecutive assays with a Bethesda assay result of 0.6 BU or more and no detectable FVIII activity assessed by FVIII activity assay below 1%. All the patients included had been treated for at least 50 days (50 days of exposure).

## Statistical analyses

**Fig 1:**
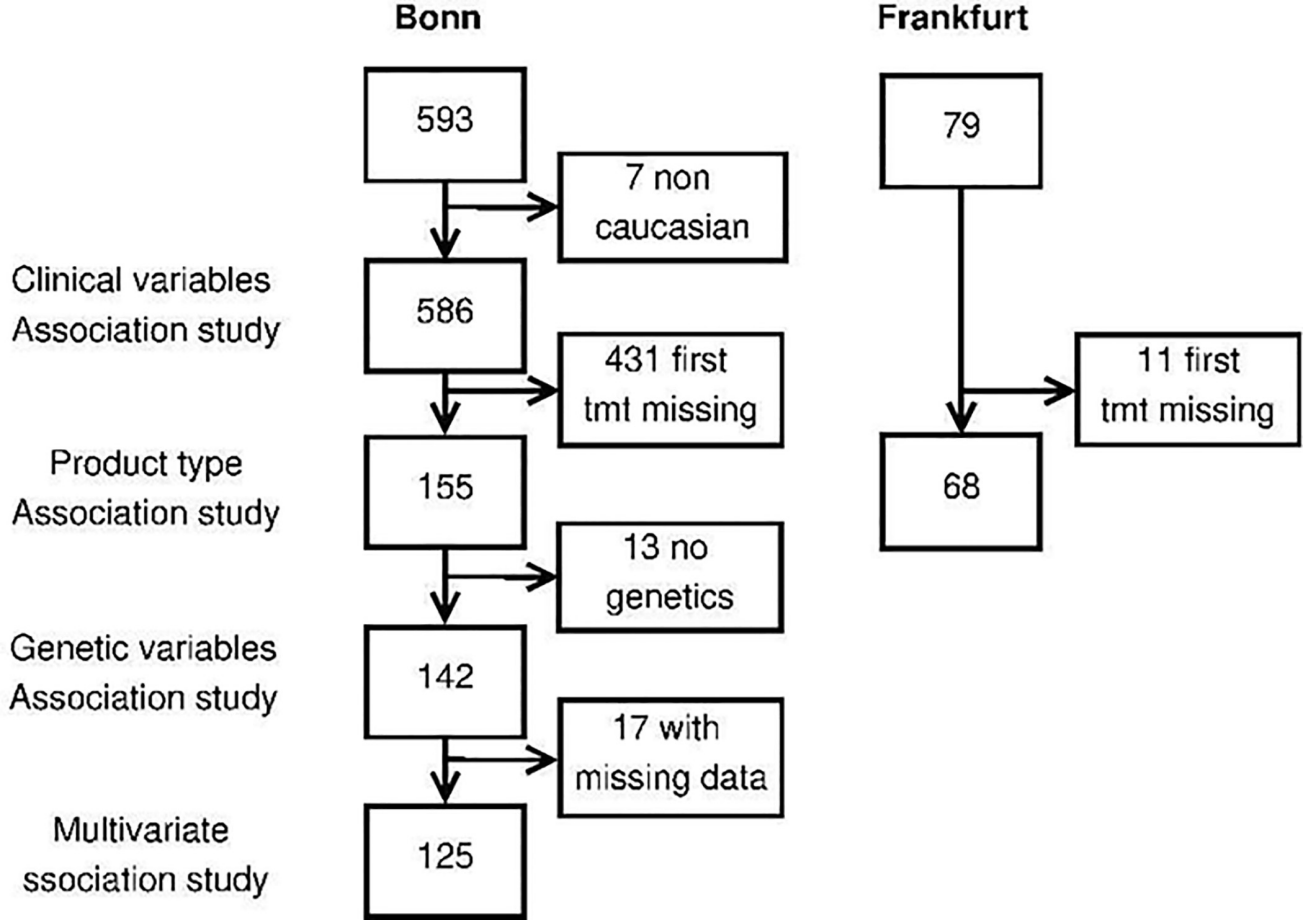
Patient inclusion flow chart per site in association studies.

The selection of patients for the different analyses is described in a flowchart in Fig 1.

In the Bonn entire cohort, people were born from 1920 to 2012. This interval was cut in three smaller intervals: before 1978, 1978–1995 and after 1995. Inhibitor positivity rates were different in these three categories.

A descriptive analysis of patient, disease and treatment characteristics for the entire dataset was performed with univariate logistic regressions taking into account birth cohort effect and “well-documented” status. To avoid blurring any association between a variable and the outcome, these analyses were run separately on Bonn and Frankfurt datasets: a first exploration was carried out on Bonn, then a confirmation on Frankfurt’s dataset. If results were consistent, analyses were performed on the pooled cohort.

For each polymorphism, genotype frequencies were determined. Descriptive analyses of genetic markers were performed with chi-square tests (or Fisher's exact test as needed) and univariate logistic regression. A classic additive genetic model was considered.

The multivariate analyses were performed only on the "well-documented" population as previously defined, in order to obtain unbiased analyses.

Two multivariate models were considered. The first model was a classic multivariate logistic regression model where both clinical and genetic factors were candidate variables. The model was constructed using a stepwise forward strategy, starting with variables having univariate p-values below 0.2, and proceeding with entry and removal criteria at p-values of 0.05.

The second model was the Generalized Partially Linear Tree-based Regression (GPLTR). This tree-based model provides a classification of patients in homogeneous groups in terms of risk of inhibitor development and identifies relevant genetic interactions. This procedure is a hybrid multivariate approach combining both GLM (generalized linear models) and CART (classification and regression trees). The GPLTR modeling procedure is described elsewhere [23]. Briefly, an iterative procedure is used in a first step to build trees that are grown to maximum size and adjusted on selected variables. A forward procedure is used in a second step to compute a set of nested subtrees. The optimal GPLTR tree is finally selected via the Bayesian Information Criterion (BIC).

As regression-trees are prone to instability, i.e. a small change in the data set can result in different series of splits, thus making variable selection somewhat precarious, we also constructed multiple trees using a bootstrapping approach [24]. This procedure is not intended to give prediction since our dataset is underpowered for reaching such objective. However, it is a way to evaluate the stability of the association results. More precisely, the bagging procedure enables computation of variable importance measures that assess the relevance of each variable across the set of trees. It provides a way to rank the variables according to their discriminative power. Variables that are associated with the outcome have large values as compared to those who are not associated with. These latter results provide us some arguments regarding the reliability of the selected optimal GPLTR tree.

Statistical analyses were performed using R software (version 3.3.1. software). Haplotype analyses were performed using the 'HaploStats' R package. Regression tree analyses were performed with the 'GPLTR' R package [25].

## Results

The dataset comprised 593 and 79 patients with severe HA from the Bonn and the Frankfurt databases respectively. Univariate analyses for well-established risk factors were performed separately. A pooled analysis was performed when results exhibited the same trend.

As shown in Fig 1, the analyses excluded 442 patients for whom we had no reliable information on the first exposure to FVIII products.

Of the 586 patients from Bonn and the 79 patients from Frankfurt, respectively 113 (19%) and 32 (41%) developed inhibitors. The analysis of birth cohort effect showed that, in Bonn, the incidence of inhibitors increased over time: 12% of the patients born before 1978 developed inhibitors, 23% between 1978 and 1995 and 44% after 1995. There was no significant difference between birth cohorts in Frankfurt (22% before 1995 and 19% after 1995).

### Univariate analysis

Patients with an F8 mutation risk considered as “high risk” had a 3.61 times greater risk (95% CI 2.13–6.40) of developing inhibitors compared to patients with a “low risk” according to the pooled analysis of the Bonn and Frankfurt data (Table 2). Among patients with a history of hemophilia A, the presence of a family history of inhibitors was associated with a 5.94 times greater risk (95% CI 2.73–13.29) of developing inhibitors in the Bonn dataset. This information was not available in the Frankfurt dataset. Patients with a blood group other than O were more likely to develop inhibitors than those with group O. This association did not reach statistical significance in the pooled dataset, with patients with blood group other than O having a 1.46 times greater risk (95% CI 0.94–2.31). As shown in the flow-chart (Fig 1), the type of FVIII at first exposure could only be analyzed in the “well-documented” subgroup of the patients. In the Bonn subgroup, patients first exposed to recombinant FVIII developed more inhibitors than those exposed to plasma-derived products (OR = 2.18, 95% CI 0.97–4.99). This association was not observed in the Frankfurt database.

**Table 2:** Univariate odds ratios for patient, disease and treatment risk factors for inhibitor development.

		Bonn (N = 586)				Frankfurt (N = 79)				Pooled (N = 665)
		inh-	inh+			inh-	inh+			Inh-
		N = 473	N = 113			N = 47	N = 32			N = 520
		n (%)	n (%)	OR* (95% CI)	p-value	n (%)	n (%)	OR** (95% CI)	p-value	n (%)
F8 mutation	Low risk	166 (37)	15 (14)	1 reference	<0.0001	15 (32)	4 (13)	1 reference	0.06	181 (36)
type	High risk	286 (63)	89 (86)	3.69 [2.04–7.07]		32 (68)	28 (88)	3.27 [1.04–12.59]		318 (64)
	Missing	21	9							21
Family history	No family history of HA	343 (73)	63 (56)	1.27 [0.72–2.31]	0.43	MISSING				
	Family history of HA without inhibitors	107 (23)	21 (19)	1 reference						
	Family history of HA and of inhibitors	23 (5)	29 (26)	5.94 [2.73–13.29] <0.0001						
Blood group	O	166 (37)	33 (31)	1 reference	0.21	15 (44)	7 (30)	1 reference	0.23	181 (37)
	Others	285 (63)	69 (73)	1.37 [0.84–2.26]		19 (56)	16 (70)	2.01 [0.65–6.59]		304 (63)
	Missing	22	7			13	9			35
Type of FVIII	Plasma derived	73 (84)	49 (72)	1 reference	0.06	29 (73)	21 (75)	1 reference	0.87	
at first exposure	Recombinant	14 (16)	19 (28)	2.18 [0.97–4.99]		11 (27)	7 (25)	0.91 [0.29–2.76]		
	Missing	386	45			7	4			

*Adjusted on birth cohorts (before 1978, 1978–1995, after 1995) and “well-documented” status

**Adjusted on birth cohorts (before 1995, after 1995) and”well-documented” status

***Adjusted on birth cohorts (before 1995, after 1995), “well-documented” status and site (Bonn or Frankfurt)

HLA class II markers and immune response genes IL-10, TNF-a, CTLA-4, CD32, MAPK9 and CD86 were analyzed for their association with inhibitor development in 142 patients from the Bonn database.

(Fig 1). We found no statistically significant deviation from the Hardy Weinberg equilibrium for any of the candidate SNPs. Table 3 gives the univariate odds ratios for each genetic factor.

**Table 3:** Univariate odds ratios for genetic risk factors of inhibitor development.

		inh- (N = 79)	inh+ (N = 63)		
		N = 79 n (%)	N = 63 n (%)	OR (95% CI)	p (chi^2^ or Fisher's test)
HLA	DRB1*01	**21 (27)**	**7 (11)**	**0.34 [0.13–0.83]**	**0.03**
	DRB1*03	10 (13)	10 (16)	1.28 [0.49–3.35]	0.78
	DRB1*04	14 (18)	14 (22)	1.31 [0.57–3.01]	0.67
	DRB1*07	17 (22)	14 (22)	1.03 [0.46–2.28]	1
	DRB1*08	10 (13)	2 (3)	0.22 [0.02–1.12]	0.07
	DRB1*11	27 (35)	15 (24)	0.59 [0.27–1.23]	0.23
	DRB1*13	22 (28)	16 (25)	0.87 [0.40–1.83]	0.86
	DRB1*15	**17 (22)**	**31 (49)**	**3.48 [1.70–7.34]**	**0.001**
	DRB1*16	2 (3)	3 (5)	1.89 [0.21–23.32]	0.66
	DQB1*02	20 (26)	18 (29)	1.14 [0.54–2.41]	0.88
	DQB1*03	43 (56)	30 (48)	0.72 [0.37–1.40]	0.42
	DQB1*04	10 (13)	2 (3)	0.22 [0.02–1.10]	0.07
	DQB1*05	24 (31)	17 (27)	0.82 [0.39–1.70]	0.72
	DQB1*06	**31 (40)**	**38 (60)**	**2.24 [1.08–4.71]**	**0.03**
	missing	1	0		
TNFalpha	G/G	54 (68)	44 (71)	1 reference	0.89
(-308 G>A)	G/A	23 (29)	16 (26)	0.85 [0.40–1.80]	
rs1800629	A/A	2 (3)	2 (3)	1.23 [0.14–10.57]	
	missing	0	1		
CTLA-4	A/A	19 (24)	12 (19)	1 reference	0.41
(CT 60)	A/G	35 (44)	35 (56)	1.58 [0.68–3.82]	
rs3087243	G/G	25 (32)	16 (25)	1.01 [0.39–2.67]	
IL-10	**G/G**	**30 (38)**	**13 (21)**	**1 reference**	**0.05**
(-1082 G>A)	**G/A**	**38 (48)**	**34 (54)**	**2.06 [0.94–4.69]**	
rs1800896	**A/A**	**11 (14)**	**16 (25)**	**3.36 [1.25–9.45]**	
HMOX1	SS	8 (10)	11 (17)	1 reference	0.28
	SM	30 (38)	25 (40)	0.61 [0.21–1.73]	
	SL	8 (10)	2 (3)	0.18 [0.02–0.96]	
	MM	20 (25)	10 (16)	0.36 [0.11–1.17]	
	ML	11 (14)	13 (21)	0.86 [0.25–2.90]	
	LL	2 (3)	2 (3)	0.73 [0.07–7.14]	
HMOX1 alleles	S	53 (34)	44 (35)	1 reference	0.96
	M	82 (52)	63 (50)	0.93 [0.55–1.55]	
	L	23 (15)	19 (15)	1.00 [0.48–2.06]	
CD32	G/G	17 (22)	14 (22)	1 reference	0.90
rs1801274	G/A	34 (43)	29 (46)	1.04 [0.44–2.48]	
	A/A	28 (35)	20 (32)	0.87 [0.35–2.17]	
MAPK9	C/C	45 (57)	37 (59)	1 reference	0.33
rs4147385	C/T	31 (39)	20 (32)	0.78 [0.38–1.59]	
	T/T	3 (4)	6 (10)	2.43 [0.60–12.15]	
CD86_pro	A/A	45 (57)	32 (51)	1 reference	0.56
rs2715267	A/C	26 (33)	21 (33)	1.14 [0.54–2.36]	
	C/C	8 (10)	10 (16)	1.76 [0.63–5.08]	
CD86_ex4	A/A	72 (91)	56 (89)	1 reference	0.67
rs2681417	A/G	6 (8)	6 (10)	1.29 [0.42–3.96]	
	G/G	1 (1)	1 (2)		
CD86_ex7	G/G	36 (46)	32 (51)	1 reference	0.82
rs1129055	G/A	35 (44)	25 (40)	0.80 [0.40–1.62]	
	A/A	8 (10)	6 (10)	0.84 [0.25–2.68]	
CD86_UTR	**G/G**	**22 (28)**	**28 (44)**	**1 reference**	**0.02**
rs2681401	**T/G**	**35 (44)**	**28 (44)**	**0.63 [0.30–1.32]**	
	**T/T**	**22 (28)**	**7 (11)**	**0.25 [0.08–0.67]**	

For HLA markers, HLA-DRB1*01 was associated with a lower risk (OR = 0.34, 95% CI 0.13–0.83) while HLA-DRB1*15 and HLA-DQB1*06 alleles were associated with a higher risk of inhibitor development (ORs = 3.48, 95%CI 1.70–7.34 and 2.24, 95%CI 1.08–4.71 respectively). The haplotype analysis showed an increased risk for the haplotypes 'DRB1*15-DQB1*06' (p = 0.002) and 'DRB1*04-DQB1*03' (p = 0.03).

For immune genes, only IL-10 and CD86 showed a significant association. IL-10-1082 G>A was associated with a lower risk of inhibitor development (OR = 3.36, 95% CI 1.25–9.45 for the A/A genotype compared to G/G). One SNP from the UTR region of gene CD86, rs2681401, was associated with a lower risk of inhibitor development (OR = 0.25, 95% CI 0.08–067 for the T/T genotype compared to G/G).

### Multivariate analysis

The variables associated with inhibitor development in univariate analyses remained statistically significant in multivariate logistic regression (Table 4). The HLA-DRB1*15 allele, which is in linkage disequilibrium with DQB1*06, was the one retained in the final model as it was the one with the strongest association with inhibitor development. The AIC (Akaike Information Criterion) for the final multivariate logistic model was of 142.31.

**Table 4:** Adjusted odds ratios for patient, disease, treatment and genetic risk factors for inhibitor development.

		inh- N = 67	inh+ N = 58	OR (95%CI)	P
F8 mutation type	Low risk	37	49	1 reference	0.0035
	High risk	30	9	4.34 (1.67–12.21)	
Family history	No fam. hist. of HA	39	29	1.51 (0.55–4.28)	0.4279
	Hist. of HA without hist. of inhibitors	24	10	1 reference	
	Hist. of inhibitors	4	19	7.19 (1.79–34.21)	0.0078
Type of FVIII at first	Plasma-derived	61	44	1 reference	0.0323
exposure	Recombinant	6	14	3.69 (1.16–13.05)	
IL-10	0	25	12	1 reference	0.0441
*A allele dominant*	1	42	46	1.93 (1.03–3.77)	
HLA-DRB1*15	0	52	29	1 reference	0.0078
	1 or 2	15	29	3.54 (1.42–9.28)	
CD86_UTR	0	21	27	1 reference	0.0344
*T allele dominant*	1	46	31	0.38 (0.15–0.91)	

**Fig 2:**
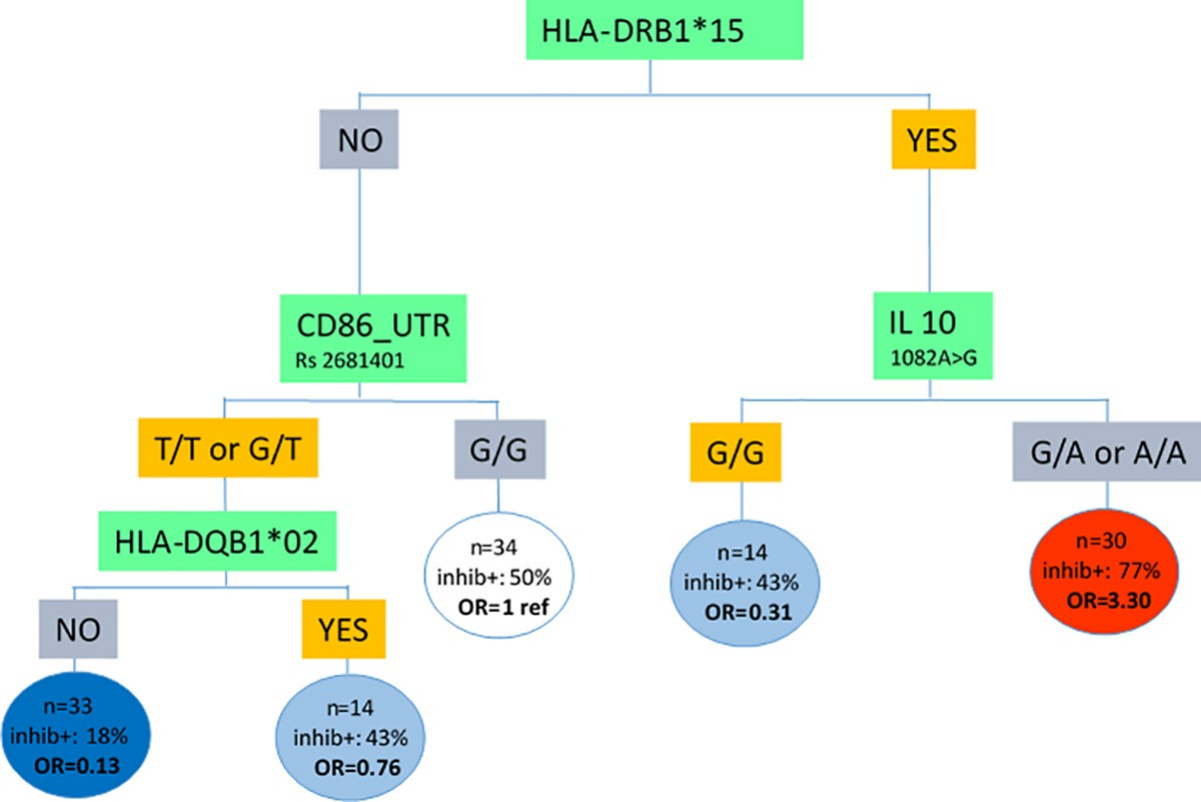
Optimal GPLTR tree associated with inhibitor development linearly adjusted on F8 mutation type, family history of inhibitors and type of FVIII product. n represents number of patients among 125 patients included in the model.

For the analyses based on the optimal GPLTR tree, the F8 mutation type, a family history of inhibitors and the type of FVIII product were included in the linear part of the model. For the tree part, all genetic factors were candidates to build the hybrid tree-based model. The final model selected the following variables: HLA-DRB1*15, CD86, IL-10, and HLA-DQB1*02 as risk factors in the tree part (Fig 2).

The optimal GPLTR tree identified three groups of patients according to their category of HLA-DRB1*15, CD86, IL-10, and HLA-DQB1*02 with probabilities of 0.18 (left branch), 0.47 (29 inhibitors positive among 62 patients in the intermediate branches) and 0.77 (right branch) of developing inhibitors. The AIC for the final model is 134.95.

Patients found negative for HLA-DRB1*15 and HLA-DQB1*02 and genotype T/T or G/T for CD86 (rs2681401) were at low risk for immunogenicity (OR = 0.13, 95% CI 0.04–0.37) whereas patients found positive for HLA-DRB1*15 and genotype G/A or A/A for IL-10 (rs1800896) were at high risk for immunogenicity (OR = 3.30, 95% CI 1.14–10.24).

**Fig 3:**
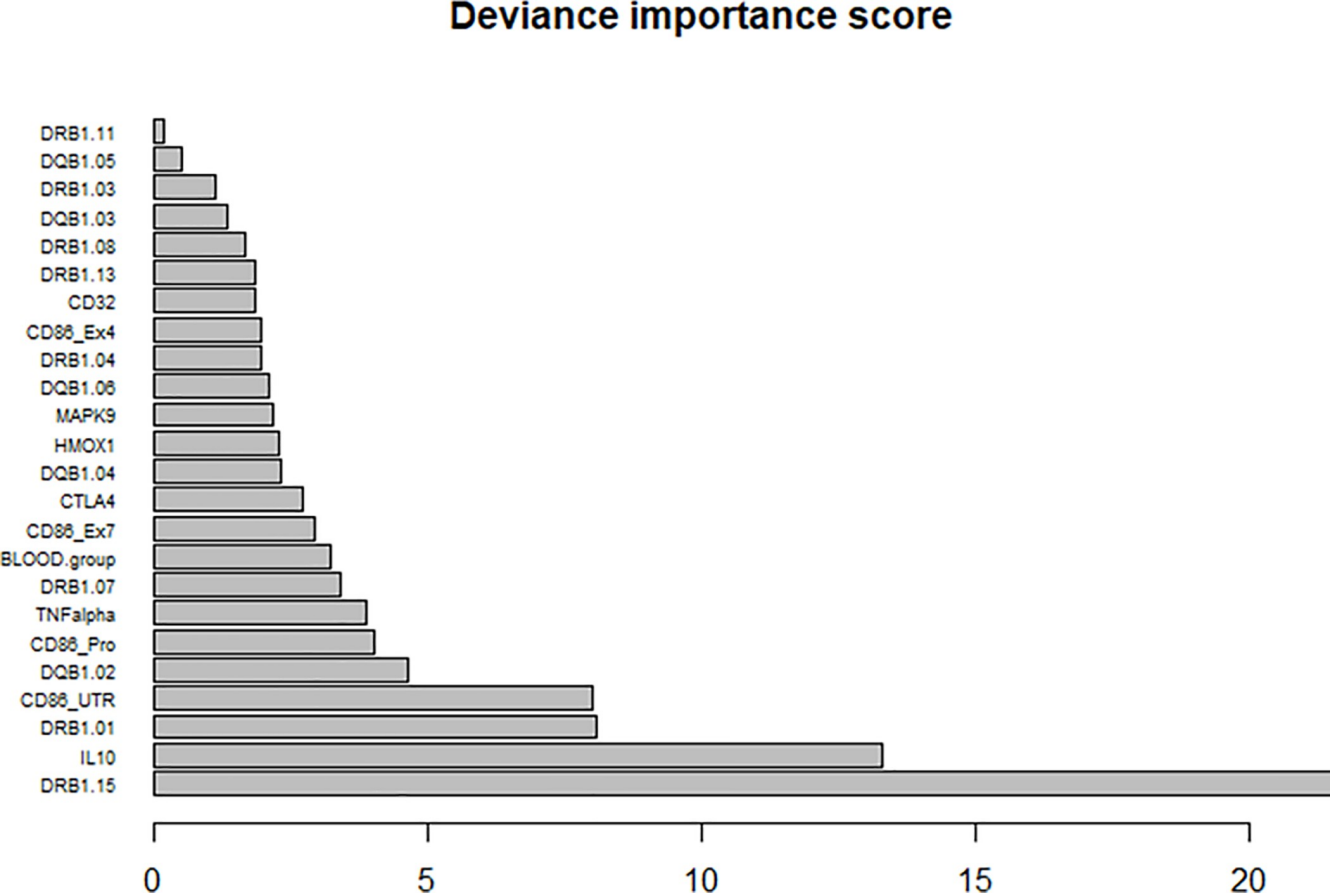
Deviance Importance Scores (DIS) obtained from bagged GPLTR for each of the competitive variables included in the multivariate model.

Results from the random forest (bagging procedure) associated with the tree-based model provide a ranking of the variables based on deviance importance scores (Fig 3). HLA-DRB1*15, IL10, CD86 and HLA-DQB1*02 which were the variables selected in the optimal GPLTR tree are among the five variables with the higher scores.

## Discussion

In this study, we investigated the association of bio-clinical and genetic markers with the development of FVIII inhibitor taking into account potential genetic high order interactions.

The classic bio-clinical factors selected by our analyses were in line with those reported in previous studies. The F8 mutation type and a family history of FVIII inhibitors were confirmed as being associated with inhibitor development in severe hemophilia A patients. The type of FVIII product (recombinant versus plasma-derived) was also selected in our analyses, this result being consistent with those of SIPPET. These bio-clinical variables were considered in the linear part of the model for both the logistic and the tree-based model. Intensity of the first treatments was not available in the dataset and could not be assessed. Taking into account these three variables, the GPLTR model identified three groups of patients according to their category of HLA-DRB1*15, CD86, IL10, and HLA-DQB1*02 with probabilities equals to 0.18, 0.47 and 0.77 to develop inhibitors.

In the multivariate logistic model, the classic categorization of F8 mutation type as “low” versus “high” risk shows an odds ratio in the same range (around four) as in the previously published methods [16]. A family history of FVIII inhibitor was also associated with inhibitor development. Interestingly, even with adjustment on genetic factors, the odds ratio remains high (around seven) suggesting that other genetic risk factors contribute to this hereditary risk.

The multivariate logistic regression model showed that HLA-DRB1*15, CD86, and IL-10 variants had an impact on the occurrence of neutralizing inhibitors. HLA-DRB1*15 has already been shown to be associated with inhibitor development [5]. In the present study, other HLA markers, such as DRB1*01, DQB1*06, were associated with inhibitor development in the univariate analysis but not in the multivariate analysis. As these markers are in strong linkage disequilibrium, the final multivariate logistic model only selected the one that made the highest contribution. Previous studies reported an association between IL-10 polymorphisms and inhibitor development [3,8–11]. We showed that IL-10 (1082A>G) is a risk factor of inhibitor development. The protein encoded by the IL-10 gene is a cytokine that has pleiotropic effects in immune regulation and inflammation. In vitro studies indicate that the 1082G allele is associated with higher IL-10 production and the A allele with lower IL-10 production [26]. We observed that the 1082A allele was associated with a higher probability of inhibitor development, suggesting that low IL-10 production is associated with higher inhibitor risk. For the first time to our knowledge, we report that one SNP in the UTR region of CD86 gene (rs2681401) was associated with a lower risk for T/T and G/T genotypes. Interestingly the tree representation suggests that these three genes, HLA-DRB1*15, IL10 and CD86, are part of a same immune cascade, with CD86 involved in activation of the antigen presenting cells [20], HLA in the presentation to T-cells and IL-10 in the immune regulation. We could not confirm any association of SNPs in the TNFalpha, CTLA4 and HMOX1 genes with inhibitors, but the sample size may be too small to detect it.

Taking into account F8 mutation type, family history of inhibitors, and type of FVIII product, the optimal GPLTR hybrid tree-based model also selected HLA-DRB1*15, CD86, and IL-10 and provided additional information by also selecting HLA-DQB1*02. Of note, based on these genetic markers, the optimal GPLTR tree provided a partition structure of the whole population under study that formed more homogeneous groups for inhibitor development. The hybrid tree-based model showed that the highest risk for immunogenicity was observed in patients with positive HLA-DRB1*15 and IL-10 genotype G/A and A/A. Of the 30 patients (24% of the total) in this group, 23 experienced FVIII inhibitors. In contrast, the lowest risk group for immunogenicity was defined by negative HLADRB1*15/ HLADQB1*02 and CD86 (TT or G/T). In this group, among the 33 patients (26% of the total), only 4 experienced inhibitors. The other groups formed by the tree-based model had an intermediate risk. Results obtained from the bagging procedure confirmed the importance of the selected variables and suggest that the final tree-based model is sufficiently reliable. It is also worth noting that the GPLTR model provided a better fit for the data according to the Akaike information criterion compared to a classic multivariate logistic regression approach.

Concerning blood group, patients in blood group O were less likely to develop inhibitors but the association was not statistically significant in the univariate analysis combining the Bonn and Frankfurt cohorts (p = 0.10). In the eligible subset of the Bonn cohort, blood group was also not significant in the multivariate analysis. This could result from a lack of power. It is worth noting that this variable is classified as the nineth in terms of fit scores by the bagged GPLTR procedure. An association between blood group and inhibitors has previously been reported [27]. Given the data on the potential role of von Willebrand factor (VWF) in immune recognition of FVIII [28] and inhibitor development [2], it is of note that levels of VWF differ in individuals with different blood groups. This association warrants further investigation.

The ABIRISK project, as a collaborative initiative, enables data from different sites to be pooled. Investigation of the main clinical factors (patient-, disease- and treatment-related) was based on the historical cohorts of Bonn and Frankfurt. Datasets were however rather different in terms of data availability, especially concerning genetic information. In addition, a selection of population in terms of homogeneity in birth cohort and follow-up was necessary. While the descriptive analyses were carried out on the whole population, a small subpopulation from the Bonn cohort was used for the multivariate analyses. Mostly patients treated previously elsewhere were excluded as some were patients treated a long time ago and as there were no reliable information on first treatment for these patients. With this unbiased but reduced population, however, comes a decrease in power for the statistical analyses. Our study has some limitations that should be mentioned. Since it is an observational study, we could not exclude some bias between negative and positive inhibitor patients, these latter been more well documented. The majority of the population are Caucasian but we could not exclude some heterogeneity in the ancestry background of our population. Moreover, our dataset has a reduced sample size and additional studies with larger sample size are required to strengthen our findings.

## Conclusion

The present study investigates the relationship between genetic factors and FVIII inhibitor development in severe hemophilia A patients, together with F8 mutation type, a family history of inhibitors and FVIII product type. It relies on a hybrid tree-based model which is well suited to investigate high-order interactions. The final optimal tree distinguishes two groups of patients: a high-risk group for immunogenicity with positive HLA-DRB1*15 and IL-10 genotype G/A and A/A, a low-risk group for immunogenicity with negative HLADRB1*15/ HLADQB1*02 and CD86 genotype T/T and G/T.
